# Dynamic Patterns of N6-Methyladenosine Profiles of Messenger RNA Correlated with the Cardiomyocyte Regenerability during the Early Heart Development in Mice

**DOI:** 10.1155/2021/5537804

**Published:** 2021-08-06

**Authors:** Yuhui Yang, Siman Shen, Yin Cai, Kejun Zeng, Keyu Liu, Simeng Li, Lanfen Zeng, Linming Chen, Jing Tang, Zhe Hu, Zhengyuan Xia, Liangqing Zhang

**Affiliations:** ^1^Department of Anesthesiology, Affiliated Hospital of Guangdong Medical University, Zhanjiang, China; ^2^Key Laboratory of Organ Functional Injury and Protection and Department of Translational Medicine of Zhanjiang, Zhanjiang, China; ^3^State Key Laboratory of Pharmaceutical Biotechnology and Department of Medicine, University of Hong Kong, Hong Kong SAR, China; ^4^Department of Health Technology and Informatics, The Hong Kong Polytechnic University, Hong Kong SAR, China

## Abstract

N6-Methyladenosine (m6A) plays important roles in regulating mRNA processing. Despite rapid progress in this field, little is known about the role and mechanism of m6A modification in myocardial development and cardiomyocyte regeneration. Existing studies have shown that the heart tissues of newborn mice have the capability of proliferation and regeneration, but its mechanism, particularly its relation to m6A methylation, remains unknown. *Methods*. To systematically profile the mRNA m6A modification pattern in the heart tissues of mice at different developmental stages, we jointly performed methylated RNA immunoprecipitation sequencing (MeRIP-seq) and RNA sequencing (RNA-seq) of heart tissues of mice, respectively, aged 1 day old, 7 days old, and 28 days old. *Results*. We identified the linkages and association between differentially expressed mRNA transcripts and hyper or hypomethylated m6A peaks in C57BL/6J mice at different heart developmental stages. Results showed that the amount of m6A peaks and the level of m6A modification were the lowest in the heart of mice at 1 day old. By contrast, heart tissues from 7-day-old mice tended to possess the most m6A peaks and the highest global m6A level. However, the m6A characteristics of myocardial tissue changed little after 7 days old as compared to that of 1 day old. Specifically, we found 1269 downmethylated genes of 1434 methylated genes in 7-day-old mouse heart tissues as compared to those in 1-day-old mice. Hypermethylation of some specific genes may correlate with the heart's strong proliferative and regenerative capability at the first day after birth. In terms of m6A density, the tendency shifted from coding sequences (CDS) to 3′-untranslated regions (3′UTR) and stop codon with the progression of heart development. In addition, some genes demonstrated remarkable changes both in methylation and expression, like kiss1, plekha6, and megf6, which may play important roles in proliferation. Furthermore, signaling pathways highly related to proliferation such as “Wnt signaling pathway,” “ECM-receptor interaction,” and “cardiac chamber formation” were significantly enriched in 1-day-old methylated genes. *Conclusions*. Our results reveal a pattern that different m6A modifications are distributed in C57BL/6J heart tissue at different developmental stages, which provides new insights into a novel function of m6A methylation of mRNA in myocardial development and regeneration.

## 1. Background

The adult human heart does not have sufficient ability to renovate the damaged cardiac cardiomyocytes (CMs), which is the critical factor leading to the high mortality of cardiovascular diseases [1]. Although many approaches are designed to repopulate the damaged CMs, like transplanting various sources of exogenous stem cells with differential potential [2, 3], these therapies have various limitations in treating myocardial infarction (MI) or heart failure efficaciously, such as immune response [4] and epigenetic influence [5, 6]. Interestingly, extensive recent studies show that targeting mechanisms that govern endogenous repair and proliferation to cardiomyocytes may prove to be a valid therapy for heart disease [7–9]. The adult mammalian heart has been traditionally regarded as an organ of terminal differentiation capability. Recent studies, however, discovered that several species, including neonatal mice, 1-day-old pigs, and adult zebrafish, could stimulate a robust regenerative response during cardiac injury [10, 11]. Unlike the adult zebrafish, the CMs of neonatal mice possess the proliferative capability and maintain the competence to renovate their damaged cardiac muscle tissue during the first 7 days of life [11, 12]. Convincing evidence shows that heart regeneration in neonatal mice is achieved by cardiomyocyte proliferation and the cardiac developmental program for self-renewal [12–14]. The underlying mechanisms of neonatal cardiac proliferation remain largely unclear, but the related research is of crucial significance for discovering therapeutic targets for cardiomyocyte regeneration and cardiac repair.

N6-Methyladenosine (m6A), the most common internal modification of messenger RNA (mRNA) and noncoding RNAs (ncRNA) in eukaryotes identified in the 1970s, is dynamically regulated by a set of enzymes classified into methyltransferases (“writers”), demethylases (“erasers”), and m6A binding proteins (“readers”) [15]. Over the last decade, several studies have characterized the m6A mRNA landscape in multiple organisms, such as mammals [16], yeast [17], and plants [18], and these studies have identified the consensus sequence RRACH (in which R represents A or G and H represents A, C, or U), which suggests the significance of m6A modification in multispecies conservatism. Thus, imbalance in m6A modification may impact on various diseases and biochemical progress, like regulating plant embryonic development [19], immune cell homeostasis and function [20], and cancer in various organs [21], and contribute to human disease heritability [22]. Transcriptome-wide analyses have shown that m6A modified over one-third of the mRNA in humans and mice [16]. The m6A possesses 1-3 modification sites in each particular mRNA that enrich in near stop codons, 3′UTRs, and RRACH sequence of mRNA. These studies also suggested that m6A modification has a crucial effect on various cellular pathways and processes, including developmental regulation, the cell cycle, fate determination, and the heat-shock stress response by regulating the splicing, expression, stability, and translation efficiency of mRNAs [23, 24].

Recently, the regulatory role of m6A in heart diseases has been increasingly recognized [25]. In addition, the effects of m6A modification on embryonic neural stem cells (NSCs) have been demonstrated during early brain development in newborn mice [26]. It follows that the function and correlation of m6A modifications in biological physiology and disease progression have become of great interest [25, 26]. Technical advances in mammalian studies, such as transcriptome-wide analysis, open up a novel method for revealing the distribution and function of this modification through the biotechnologies of RNA-seq, RIP-seq, and m6A-seq. To date, however, study about the m6A modification of mRNAs in mammals' myocardial proliferation is rare. And researches focusing on the development of therapies that may stimulate myocardial regeneration by mining and interfering related regulating molecules with different m6A modification during cardiac development are lacking.

The proliferative ability of mouse cardiomyocytes can only be maintained for a short period after birth. The ability of DNA synthesis is an intuitive index to reflect proliferation ability, and the activity of the enzymes needed for DNA synthesis in mice decreases significantly to the level of adult at about one week after birth [27, 28]. Current researches indicated that the heart of mice aged up to 7 days old has the ability to proliferate and regenerate, while hearts from 28-day-old mice could hardly proliferate and regenerate which is similar to that seen in adult hearts [12, 29]. So most researchers usually choose 1-day-old, 7-day-old, and 28-day-old mice (hereafter referred to as P1, P7, and P28) to study the phenomenon and mechanisms of myocardial regeneration [30, 31]. We hypothesized that m6A might play a significant role in regulating and affecting the development and regeneration of rodent hearts. Thus, in the present study, we conducted an m6A-specific analysis and bioinformatics analysis in mRNAs of mouse hearts at the three stages, including P1, P7, and P28, in an effort to provide clinical and therapeutic insights and reveal the role and mechanism of m6A in myocardial development.

## 2. Materials and Methods

### 2.1. Animal Studies

The study protocol was reviewed and approved by the animal care committees of both Southern Medical University and Guangdong Medical University. Male C57BL/6J mice were randomly assigned to three groups according to different ages (P1, P7, and P28). Groups P1 and P7 consisted of 12 animals, respectively, and group P28 consisted of 3, providing 3 biological replicates to be analyzed. Given that the heart sizes for animals in groups P1 and P7 were small, the samples from every 4 hearts were pooled for analyses. All mice in each group were deep anesthetized with ketamine (80 mg/kg, IP.) + xylazine (10 mg/kg, IP.) and were executed by cervical dislocation. Subsequently, the cardiac tissues were collected and frozen in liquid nitrogen at -80°C for further RNA extraction. Animals were obtained from the Animal Research Center of Southern Medical University. The Guide for the Care and Use of Laboratory Animals and Animal Welfare Act are followed to guide 3M's animal research program.

### 2.2. Total RNA Preparation

RNA isolation was performed with Trizol Reagent (Thermo Fisher Scientific, Waltham, MA, USA) according to the manufacturer's instructions. The ratio of OD260/280 to OD260/230 of the product was detected by NanoDrop (Thermo Fisher Scientific, Waltham, MA, USA) as the sample purity index. And the degree of RNA degradation was detected by agarose gel electrophoresis and Agilent 2100 Bioanalyzer (Agilent, Santa Clara, CA, USA). If the OD260/280 value was between 1.8 and 2.2, OD260/230 ≥ 2.0, and RIN ≥ 7, the RNA purity and integrity were qualified and marked as “Pass”.

### 2.3. RNA Purification and Fragmentation

The rRNA probe with specific species (mouse) was incubated with total RNA, and then, the captured rRNA probe was modified with biotin (Thermo Fisher Scientific, Waltham, MA, USA). The magnetic beads (Thermo Fisher Scientific, Waltham, MA, USA) coated with streptavidin were combined with the probe-rRNA complex to remove rRNA. After another purification of AMPure XP magnetic beads (Beckman Coulter, Brea, CA), the RNA without rRNA was extracted. The purified RNA was diluted in fragmentation buffer for elution, fragmentation, and random primers; then, the product was incubated at 94°C for thermal fracture and lysed into fragments between 100 and 300 bp.

### 2.4. cDNA Library Construction and Sequencing

The fragmented RNA was divided into two parts. One part was added with premixed m6A antibody immunomagnetic beads to enrich the m6A methylated mRNA fragments. Then, the enriched m6A antibody immunomagnetic beads and the recovered m6A-containing mRNA fragment were used to construct a conventional sequencing library according to the transcriptome library construction process. The other part was used as a control to construct a conventional transcriptome sequencing library directly. These two sequenced libraries, m6A-seq library and RNA-seq library, were sequenced with high throughput, respectively.

### 2.5. Sequencing Data Analysis

Libraries were sequenced and visualized on Illumina NovaSeq™ 6000 (Illumina, San Diego, CA). First, the software Cutadapt and local Perl scripts removed the low-quality, contaminated, and sequencer connector sequences to obtain clean data [32]. Next, Fastp was used to perform quality control on clean data. Then, the reads were aligned to the referential genome using the default parameters of HISAT2 [33] and peak calling analysis and peak annotation were performed by ExomePeak and ChIPseeker [34]. After that, Homer (or MEME) was applied to perform motif analysis on enriched sites and StringTie to perform transcriptome analysis and gene quantification. Finally, the R package “Edge R” was used for genetic difference analysis [35].

### 2.6. GO and KEGG Pathway Database Analysis

We used Gene Ontology (GO; http://www.geneontology.org/) to perform functional enrichment and applied GO annotation to describe the functions of the differentially methylated genes, which were classified into three major categories: biological process (BP), cellular component (CC), and molecular function (MF). In the meantime, Kyoto Encyclopedia of Genes and Genomes (KEGG; http://www.kegg.jp/) analysis was also conducted and the major terms of signal transduction pathways and biochemical metabolic pathways were identified that participated for the DEGs. When the corrected *P* value was less than 0.05, the GO terms and KEGG analysis were regarded as significantly enriched, as previously described [36, 37].

### 2.7. Quantitative Real-Time PCR

Q-PCR was performed on Roche LightCycler 480 system (Roche Applied Science, IN, USA). Relative expressions of genes were compared by the 2-*ΔΔ*Ct method, and GAPDH served as the internal housekeeping gene. The sequences of all the specific primers were designed to span extron-intron to prevent the improper amplification of mRNA. The primer sequences were as follows: IGF2BP1, 5′-GGCGACTCATTGGCAAGGAAGG-3′ (forward) and 5′-TGAGGTCCTGGAGCGATGAGATG-3′ (reverse); IGF2BP3, 5′-CATCTGTTTATTCCCGCCCTGTCC-3′ (forward) and 5′-TCACCATCCGCACTTTAGCATCTG-3′ (reverse); ALKBH5, 5′-TTCTTCAGCGACTCGGCACTTTG-3′ (forward) and 5′-CGGCAGAGAAAGCACAGGTTCC-3′ (reverse); Hist1h2ao, 5′-GCTCCGCAAGGGCAACTACTC-3′ (forward) and 5′-CCCGCCAGCTCCAGGATCTC-3′ (reverse); Tet2, 5′-CTGCTGTTTGGGTCTGAAGGAAGG-3′ (forward) and 5′-GTTCTGCTGGTCTCTGTGGGAATG-3′ (reverse); GATA4, 5′-CGAGATGGGACGGGACACTACC-3′ (forward) and 5′-TGGCAGTTGGCACAGGAGAGG-3′ (reverse); and MEGF6, 5′-TGCGACCCTGAGACTGGAACC-3′ (forward) and 5′-TTGGCACAAGCACACCTCATCTG-3′ (reverse).

### 2.8. Western Blot

Proteins were harvested and dissolved in RIPA lysis buffer, and protein concentrations were detected by enhanced Bicinchoninic Acid (BCA) protein assay kit (Beyotime, China). And the equivalent amounts of protein were separated by SDS-PAGE on 10% acrylamide gels at 60 v for 2.5 h and transferred to PVDF membranes under a constant current of 340 mA for 1.5 h. Quantitative analysis was performed by ImageJ. The primary antibodies were anti-ALKBH5 (ab195377, Abcam, MA, USA), anti-METTL3 (86132S, CST, MA, USA), anti-YTHDF1 (ab252346, Abcam, MA, USA), anti-IGF2BP3 (ab179807, Abcam, MA, USA), anti-FTO (31687S, CST, MA, USA), and anti-GAPDH (ab8227, Abcam, MA, USA). The secondary antibodies were goat polyclonal anti-Rabbit-IgG (14708S, CST, MA, USA).

### 2.9. Statistical Analysis

One-way ANOVA with Tukey's post hoc tests was carried out for comparison of multiple groups. All experiments were performed at least three times independently. Data are shown as mean ± S.D. A *P* value less than 0.05 was considered statistically significantly different.

## 3. Results

### 3.1. General Features of Cardiac m6A Methylation in Mouse during Heart Development

**Figure 1:**
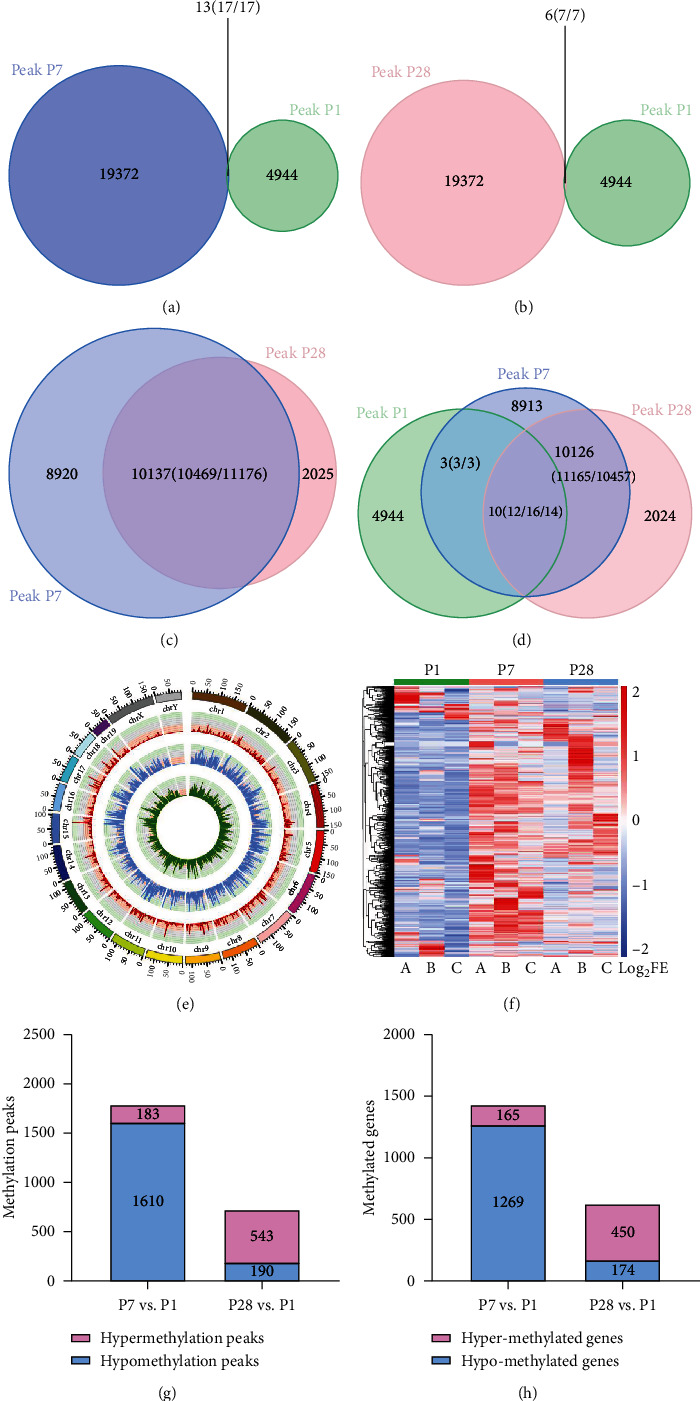
General features of m6A methylation in mouse heart development. (a) Venn diagram of m6A peaks in P7 and P1 heart tissues. (b) Venn diagram of m6A peaks in P28 and P1 heart tissues. (c) Venn diagram of m6A peaks in P28 and P7 heart tissues. (d) Venn diagram of m6A peaks in all 3 groups. (e) Distribution of m6A methylation sites on chromosome by Circos plot. Red represents P1, blue represents P7, and green represents P28. (f) Cluster analysis of m6A in P1, P7, and P28 heart tissues. The color represents the degree of the log fold enrichment (FE) value: the larger the LogFE value, the closer the color is to red (*P* < 0.05). (g) Histogram showing the methylation peaks. P7 possessed more peaks, but the levels of methylation were mostly decreased. (h) Histogram showing the methylation peaks' corresponding genes. Same as the methylation peaks, P7 genes were mostly hypomethylated as compared to P1.

In order to investigate the potential targets contributing to heart development in C57BL/6J mice, we performed m6A sequencing to compare the gene methylation profile grouped by P1, P7, and P28. We mapped up to 4961 methylation peaks in 3062 annotated genes of P1 heart tissues, 19389 peaks in 7404 annotated genes of P7, and 13201 peaks in 5712 annotated genes of P28, respectively (all *P* < 0.05, Log_2_FC > 1). Then, we calculated their pairwise intersection by using Venn diagram. Some redundancy data would be merged in statistical mapping. For instance, suppose group A was compared with group B and one peak in A may overlap with two or more peaks in B, which was called redundancy, so the number inside the parentheses in the overlap of the Venn diagram is the total amount of intersections that actually occurred, while the number outside the parentheses indicates the amount of intersections after duplicates are excluded. Of the 4961 methylation peaks in P1, only 13 reappeared in P7, and still fewer peaks (6 peaks) reappeared in P28. However, up to 10137 methylation peaks overlapped between P7 and P28 methylation peaks (Figures 1(a)–1(c)). In general, we found that 10 specific methylation peaks simultaneously appeared among P1, P7, and P28 mouse heart tissue (Figure 1(d)). There were noticeable differences in the number of m6A peaks in either P7 or P28 as compared to P1, while this characteristic difference was inconspicuous between P7 and P28; thus, we could presume that the m6A modification was significantly altered in early heart development, but tended to stabilize after day 7.

When using Circos software to analyze the distribution of mRNA m6A peaks on the chromosomes, it was found that the distribution and number of m6A peaks on each chromosome were diverse among P1, P7, and P28' s mouse heart tissue, with the diversity on chromosome 3 being the most apparent (Figure 1(e)). The results revealed that the methylation level of whole genome was significantly upregulated when cardiomyocytes developed to P7 and then dropped to medium levels at P28. Furthermore, the autosomes in the mouse heart of each age group were more profoundly methylated compared with the sex chromosomes. Interestingly, the methylation degree of sex chromosome Y was barely noticeable.

### 3.2. Cluster Analysis

The analysis of the methylation cluster and heat map showed that the methylation differences could obviously be distinguished from each group: there were marked differences among the groups but there existed relative consistencies within the groups (Figure 1(f)). To sum up, the peaks of methylation in the P1 heart tissues were the lowest, while those in the P7 heart tissues were the highest and reduced over time. By P28, the peaks of methylation were reduced as compared to P7 but they remained high as compared to P1. In total, 1610 of 1793 methylation peaks in P7 were detected as hypomethylation, and 543 hypermethylation peaks of 733 methylation peaks in P28 were identified (Figures 1(g) and 1(h), all *P* < 0.05, Log_2_FE > 1). By comparison, more hypomethylation peaks were seen in P7 heart tissues. However, 1793 methylation peaks with significant differences accounted for only approximately 8% of the methylation peaks in P7. The rest of the peaks were unique to P7, but not in P1. This interesting trend of methylation over time suggests that specific methylation sites may have an underlying network with the strong proliferative and regenerative capability of the heart at the very first day after birth, and subsequent studies are needed to investigate the mechanism.

### 3.3. Motif Analysis

**Figure 2:**
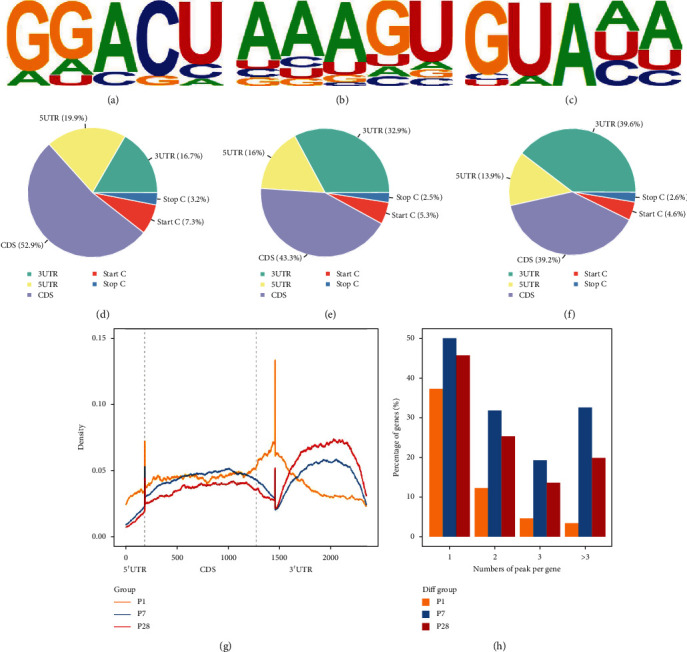
The profiles of m6A peaks and the joint analysis of m6A-seq and RNA-seq. (a–c) Motif with maximum *P* value of m6a in the P1, P7, and P28 heart tissues. (d–f) Pie chart of m6A peaks in different regions of P1, P7, and P28 heart tissues mRNA. (g) The m6A density distribution of P1, P7, and P28 heart tissues. All groups appeared at 3′UTR mostly. (h) The number of m6A peaks in P1, P7, and P28 heart tissues on each mRNA. Only one methylation peak appears among most mRNAs in each of the three groups.

While mapping the m6A methylome motif by scanning the peaks, we found that RRACH was a conserved sequence motif for m6A-containing regions among all the 3 groups, which is consistent with previous studies [11, 24]. GGACU (*P* = 1*e* − 93), AAAGU (*P* = 1*e* − 87), and GUAAA (*P* = 1*e* − 63) were the most common and reliable among the motifs in P1, P7, and P28 heart tissues, respectively (Figures 2(a)–2(c)). We, thus, speculated that difference in motifs might be one of the factors that caused m6A differences.

### 3.4. Analysis of Regions of mRNA Methylation in Different Developmental Stages of Heart Tissues

The analysis of the regions of mRNA methylation peaks showed that m6A was distributed in all regions of the mRNA (Figures 2(d)–2(f)). We observed that m6A was mostly distributed at the CDS region and 3′UTR near the stop codon in each group, which is suggestive of the direction of translational regulation, as previously described [16, 38]. Furthermore, we found that with the progression of heart development, the distribution of m6A in 5′UTR (P1: 19.9%, P7: 16%, and P28: 13.9%), CDS (P1: 52.9%, P7: 43.3%, and P28: 39.2%), and start codon (P1: 7.3%, P7: 5.3%, and P28: 4.6%) decreased gradually, but increased at 3′UTR (P1: 16.7%, P7: 32.9%, and P28: 39.6%). Nevertheless, the stop codon remained the lowest distribution in each group of heart tissues (P1: 3.2%, P7: 2.5%, and P28: 2.6%) (all *P* < 0.05, Log_2_FC > 1.5). As shown in Figure 2(g), the m6A density shifted to 3′UTR and stop codon regions from P1 to P28. These findings are consistent with previous research in Homo sapiens' mRNA methylation characteristic between neonate and adult [39], indicating that the m6A landscape of humans and mice was highly homologous.

### 3.5. Density Distribution of m6A Peaks across mRNA Transcripts

In each of the three groups, most of the m6A-modified mRNAs contained only one m6A peak, whereas a small number of them contained two or more peaks (Figure 2(h)), which was in accordance with previous studies [38]. And this characteristic in the P7 heart tissues was particularly high (~50%, *P* < 0.05). Likewise, the quantities of mRNAs with two or more m6A peaks were the largest in day 7 heart tissues.

### 3.6. Effect of RNA m6A Modifications on Gene Transcriptional Expression

**Figure 3:**
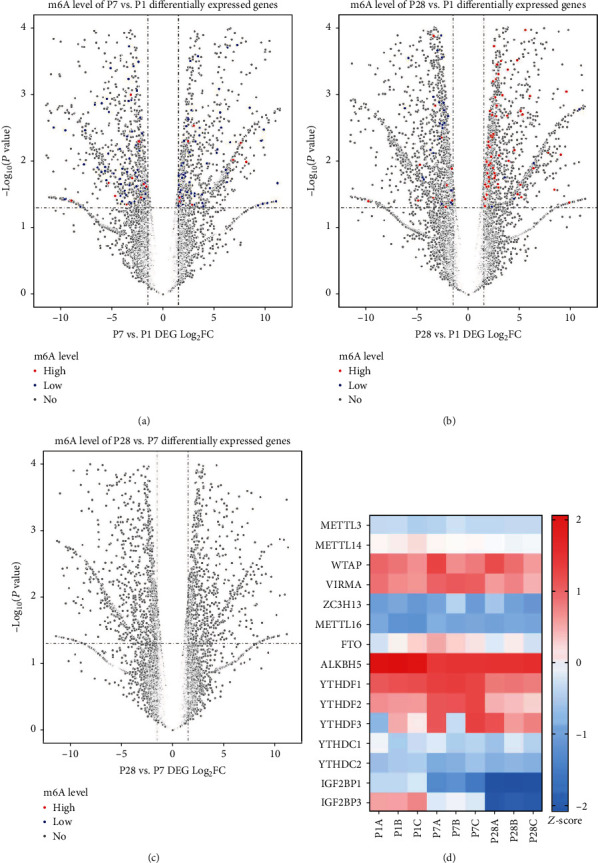
Joint analysis of gene transcriptome and methylation. (a) Different gene expressions combining with methylation level in P7 and P1 heart tissues. (b) Different gene expressions combining with methylation level in P28 and P1 heart tissues. (c) Different gene expressions combining with methylation level in P28 and P7 heart tissues. (d) Heat map of a series of methylase expression (*P* < 0.05, −2 ≤ *Z*‐score ≤ 2).

We performed a joint analysis of the gene transcriptome and methylation (all *P* < 0.05, Log_2_FC > 1). The result demonstrated that there were more upregulated methylated mRNAs in the P28 heart tissues than in P1 and more downregulated methylated mRNAs in P7 than in P1 heart tissues (Figures 3(a) and 3(b)). Among the 450 P28 heart tissue genes that were upmethylated, 162 genes were upregulated and 50 genes were downregulated as compared to P1. Among the 1269 genes that were downmethylated in P7 heart tissues, a total of 246 genes were upregulated and 194 genes were downregulated as compared to P1. However, barely any methylation difference appeared in DEGs between P7 and P28 (Figure 3(c)). The differences in the transcription level of hyper- and hypomethylation peaks and their corresponding genes are shown in Tables 1–4. Moreover, we noticed that some genes that were highly expressed, respectively, in both the P1 and the P7 heart tissues were rarely expressed in the P28 heart tissues, such as plekaha6 and megf6. Taken together, it may indicate that m6A-modified genes tended to have a positive regulation of expression in developing heart tissues, but further validation is required to verify this hypothesis.

Furthermore, we performed a heat map by *Z*-score analysis (*P* < 0.05) on the expression of various methylases in different developmental stages of mouse hearts. The result showed that there was no significant difference in the expression of “writers” at different developmental stages. By contrast, in “erasers,” the expression of ALKBH5 was more abundant than that of FTO, the expression of ALKBH5 was higher at P1 than at P7 and P28, and IGF2BP3 was the most highly differentially expressed “reader” protein and it decreased gradually from P1 to P28. These findings implied that “eraser” and “reader” seem to play a more important regulatory role as compared to “writers” in the development of mouse heart, especially when some methylases are highly expressed at P1. According to previous reports, the mouse heart at P1 has the strongest proliferative and regenerative capability [12], but whether these methylases highly expressed in P1 mouse heart tissues could regulate the proliferation and regeneration of mouse heart needs further work to explore.

### 3.7. Bioinformatics Analysis of Functional Genomics

Given that the 7^th^ day of heart development is being considered a watershed in myocardial regeneration and proliferation, we divided the results into two parts for bioinformatics analysis as follows.

#### 3.7.1. P7 Compared to P1

**Figure 4:**
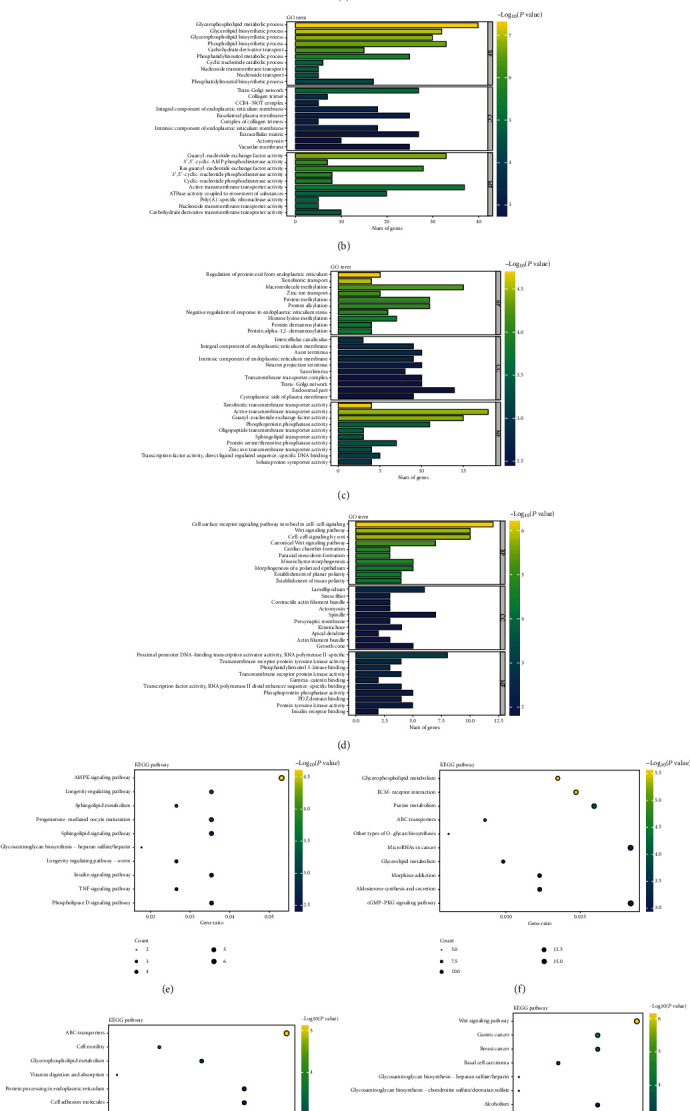
Gene Ontology and KEGG analysis of comparing C57B/L different day-old heart tissues. (a, b) Biological processes (BP), cell component (CC), and molecular functions (MF) of hyper- and hypomethylated genes in P7 as compared to P1. (c, d) Biological processes (BP), cell component (CC), and molecular functions (MF) of hyper- and hypomethylated genes in P28 as compared to P1. (e, f) KEGG pathway analysis of hyper- and hypomethylated genes in P7 as compared to P1. (g, h) KEGG pathway analysis of hyper- and hypomethylated genes in P28 as compared to P1.

For the GO terms of BP category, genes with hypermethylated m6A sites were significantly enriched in the “intracellular protein transmembrane transport,” “negative regulation of cytosolic calcium ion concentration,” and “vesicle-mediated transmembrane transport,” while hypomethylated genes were highly enriched in “glycerophospholipid metabolic process” and “phospholipid biosynthetic process.” For the CC part, genes with hypermethylated m6A sites were mainly related to “Golgi stack” and “nuclear transcriptional repressor complex,” while hypomethylated genes were primarily enriched in “trans-Golgi network” and “extracellular matrix.” As for the analysis of MF, it revealed that the “transmembrane receptor protein tyrosine kinase activity,” “ATP-dependent helicase activity,” and “ligand-gated cation channel activity” were mostly enriched in genes with hypermethylated m6A sites, while the hypomethylated genes were primarily enriched in “guanyl-nucleotide exchange factor activity” and “active transmembrane transporter activity” (Figures 4(a) and 4(b), all *P* < 0.05).

KEGG pathway analysis revealed that the most significantly overrepresented pathways among upmethylated transcripts were the “AMPK signaling pathway,” “longevity regulation pathway,” and “insulin signaling pathway” (Figure 4(e), *P* < 0.05), while the downmethylated mRNAs were significantly enriched in “glycerophospholipid metabolism,” “ECM-receptor interaction,” and “microRNAs in cancer” (Figure 4(f), *P* < 0.05).

#### 3.7.2. P28 Compared to P1

The GO analysis of BP showed that the genes with hypermethylated m6A sites were significantly enriched in “regulation of protein exit from endoplasmic reticulum,” “macromolecule methylation,” and “protein methylation,” while the hypomethylated genes were significantly detected in “cell surface receptor signaling pathway involved in cell-cell signaling,” “Wnt signaling pathway,” and “cardiac chamber formation.” The CC part indicated that the genes with hypermethylated m6A sites were mainly related to “intercellular canaliculus” and “integral component of endoplasmic reticulum membrane,” and the hypomethylated genes were mostly related to “lamellipodium” and “contractile actin filament bundle.” The analysis of MF revealed that the genes with hypermethylated m6A sites were mostly enriched in the “active transmembrane transporter activity,” “transcription factor activity,” and “direct ligand-regulated sequence-specific DNA binding,” whereas the hypomethylated genes were primarily related to “proximal promoter DNA-binding transcription activator activity,” “RNA polymerase II-specific,” and “transmembrane receptor protein tyrosine kinase activity” (Figures 4(c) and 4(d), all *P* < 0.05).

Pathway analysis revealed that upmethylated transcripts were involved in “ABC transporter,” “cell motility,” and “cell adhesion molecules” (Figure 4(g), *P* < 0.05), whereas downregulated transcripts included “Wnt signaling pathway,” “gastric cancer,” and “alcoholism” mostly (Figure 4(h), *P* < 0.05).

To sum up, there were differences between the results of these two parts. What mainly be methylated in the P7 heart tissues were genes governing cell-to-cell transport and signal transduction, while in the P28 heart tissues were genes more related to the function of macromolecules such as organelle formation. However, the overlaps that were identified in both parts, such as the “ABC transporters” and “ECM-receptor interaction pathway” of KEGG, most likely play significant roles in myocardial development process.

### 3.8. Verification of Significant Genes and Proteins

**Figure 5:**
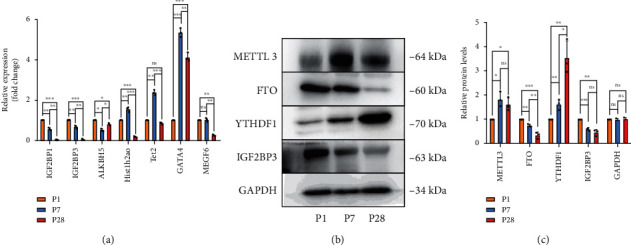
Verification of significant genes. (a) qPCR result of noteworthy methylases and genes in mouse heart tissues. (b, c) Western blot showing the representative methylases in mouse heart tissues, quantitated by ImageJ. Values are the mean ± S.D. of *n* = 3 independent experiments. ^∗^*P* < 0.05; ^∗∗^*P* < 0.01; ^∗∗∗^*P* < 001; ns: not significant.

On the basis of the m6A methylation analysis results described above, we further validated at the gene or protein levels of the major m6A methylases and genes with noteworthy differences both in methylation and expression using qPCR and western blot analysis. The qPCR results showed significant increases of GAT4and Tet2, but decreases of IGF2BP3and MEGF6 (Figure 5(a)), which were in general in agreement with the findings obtained using gene profiling. Similarly, western blot results showed that the expression of “writer” METTL 3 was upregulated in the P7 heart tissues and then dropped down in the P28 heart tissue, but still higher than that in the P1 heart tissue, which was consistent with the distribution of m6A methylation sites on the chromosome (Figures 5(b), 5(c), and 1(e)). The high expression of “eraser” FTO may exert synergistic action on the hypomethylation in P1. However, YTHDC1 and IGF2BP3, as the representative of “YTH” and “IGF” family of “reader,” changed differently over time from P1 to P28, with increased YTHDC1 and decreased IGF2BP3 (Figures 5(b) and 5(c)), which may be related to the regulation of different downstream target proteins at different times.

## 4. Discussion

m6A, a dynamic and reversible modification among different species, is one of the most common and functional RNA modifications [16–18, 40, 41]. More and more studies showed that m6A RNA methylation plays a critical role in regulating RNA metabolic processes, which was increasingly found in mammalian failing hearts and hypoxic cardiomyocytes [31, 42, 43]. In the current study, we reported the m6A distribution and differences of mRNA in C57BL/6J hearts over time during the early development stages by illustrating global m6A modification patterns and analyzing gene expression, function, and related pathways.

We revealed that the pattern of m6A modification in the neonatal mouse heart tissue was distinct from that of the adult mice. Interestingly, in the P1 heart tissues, the amount and level of global m6A peaks detected were the lowest. But when compared with the P7 heart tissues in which it possessed the largest amount and highest level of m6A peaks, it was found that most of the peaks in P1 were hypermethylated. These specific and critical genes with aberrant hypermethylation against the globally low m6A modification tendency in P1 may be the key why neonates possess the efficient proliferative and regenerative capability. Thus, we presumed that some hypermethylated genes functioned at P1 and then misfunctioned after demethylation over time. By analyzing different methylated transcripts and peaks, various biological processes and pathways that control cardiomyocyte proliferation, such as the Wnt signaling pathway [44], were found to be significantly enriched, suggesting that there exists a relationship between abnormal m6A modification and cardiac tissue regeneration and proliferation. The global change of m6A modification spectrum may be caused by the abnormal expression of m6A key enzymes [40, 45]. According to the result of the RNA-seq, differences in the expression levels of “writers,” “erasers,” and “readers” of m6A do exist in the heart tissues from mice at different days during their early life stage.

Previous studies have shown that the proliferation and regeneration ability of heart tissue basically disappears around the 7^th^ day of development, and the cell cycle of cardiomyocytes no longer exists, with only a very small proliferation rate remaining [12–14]. The underlying mechanism is still unclear. It is the terminal differentiation and nonrenewability of myocardial tissue in adulthood that prevent the heart from proliferating and repairing itself after the heart injuries, such as ischemia reperfusion injury (IRI) and MI [10–14]. The response of the mammalian heart to MI and the concomitant cell death is the substitution of fibroblasts, extracellular matrix reticulum, and proliferating cells for the damaged myocardium, thereby the scar-forming, called ventricular remodeling [46, 47]. Extensive cell therapy studies have used embryonic stem cells (ESC) and induced pluripotent stem cells (iPSC) as exogenous sources of new cardiomyocytes for treatment [9, 48]. After being transplanted into the infarcted heart, they can play a beneficial role in the repair of local and global cardiac regeneration function [49]. However, before being used clinically, some potential adverse effects need to be resolved, such as unstable phenotypes [50], low efficient cardiomyocyte transformation rate [51], tumorigenic potential [52], and arrhythmia and rejection [53]. Therefore, reactivation of the cell cycle of cardiomyocytes and stimulation of their proliferation capacity seem to be the most direct approach. Available data and results provide various strategies to trigger this process and generate new cardiomyocytes, such as the direct transdifferentiation mediated by small molecules, transcription factors, and ncRNAs. The modification of m6A has been found to be extremely important for sperm development in existing studies [54]. When the “reader” protein is inactivated, the level of m6A RNA modification mediated by it decreases significantly, leading to a significant imbalance in the posttranscriptional translation efficiency of related genes that regulate the fate determination of spermatogonial stem cells and sperm formation. It has also been shown that “writer” METTL14 plays a major role in endothelial cell inflammation induced by tumor necrosis factor-*α* [55]. METTL14 induces endothelial cell inflammation and atherosclerotic plaque by enhancing the m6A modification of FOXO1 and promoting its expression. Meanwhile, the latest studies have shown that m6A can regulate cardiomyocyte renewal [31] and the “eraser” ALKBH5 can regulate the proliferation of cardiomyocytes by demethylating YTHDF1 [56]. The findings of these studies strongly indicate that m6A is important for heart development and can promote the proliferation and differentiation of stem cells in heart disease. In our current study, in addition to explore the regulatory role of m6A modification on gene expression during heart development, a more important purpose is to find a potential therapeutic target for IRI or MI. We thus focused on analyzing and identifying the key genes related to proliferation regulated by m6A modification by comparing heart tissues at different day-ages during development with the potential to stimulate them to arousing myocardium proliferation after IRI and MI injury to achieve the therapeutic purpose. Our results show that, from the peaks and clusters of the three groups of samples, m6A modification tends to be positively correlated with mRNA expression, consistent with some previous observations [57, 58], but differed from other studies [24, 59]. This discrepancy may be due to the differences in animal tissue sources and sample collection, but it still has significant implications. In addition, the specific role of m6A modification in gene expression depends to a large extent on the downstream function of the m6A “readers” [60–62], which have been reported to affect multiple aspects of target RNA metabolism by recognizing different m6A regions, including RNA localization, splicing, transport, translation, and stability [63]. Knocking down or overexpressing key methylases may be a good strategy for studying m6A methylation-mediated cellular responses. Furthermore, the m6A-seq and mRNA-seq data collectively show that there are genes regulated by m6A in a positive or negative manner (Tables 1–4, Log_2_FC > 1, *P* < 0.05). Besides the methylases, we found that there were remarkable changes in Tet2, GATA4, and MEGF6 in our sequencing results, which were associated with proliferation in other tissues such as the lung and colorectum as shown in other studies [64, 65]. We speculate that these genes with specific m6A modification may also be capable of promoting myocardial proliferation and regeneration which deserve further study. By integrating the latest nanotechnology, m6A modification may provide a new direction for treating heart diseases [66, 67].

## 5. Conclusions

In summary, our research reveals differences in m6A of mRNA of C57BL/6J heart tissue at different developmental stages and shows the distribution and possible function of m6A through statistical analysis and thorough bioinformatics analysis. Our studies have provided a fundamental contribution for further researches aimed at identifying novel therapeutic strategy for heart IRI and MI. However, additional studies are necessary not only to understand the underlying mechanism of m6A in the myocardial regeneration but also for the therapeutic implication for cardiovascular problems.

## Figures and Tables

**Table 1:** Top ten upmethylation peaks and their corresponding genes (P7 *vs.* P1).

Chromosome	TxStart	TxEnd	Gene name	Fold change
4	88760543	88760643	**Gm13283 ↑**	349.8
13	21810736	21810849	**Gm19658 ↑**	38.3
16	17209752	17209927	**Rimbp3**	15.3
13	21810739	21810864	**Hist1h2ao**	13.9
3	96240117	96240167	**Gm20632**	9.1
3	96240098	96240173	**H2ac19**	8.5
17	47468660	47468760	**AI661453 ↑**	7.9
7	83935631	83935681	**Cemip ↓**	7.1
7	109752344	109752444	**4930431P19Rik ↑**	6.9
7	131341694	131341794	**2310057M21Rik**	5.5

Top ten upmethylation peaks and their corresponding genes (P7 *vs.* P1). Fold change represents methylation peak's degree. Arrow represents up or down regulation of gene expression, Log_2_FC > 1.5, *P* < 0.05 (no arrow means no statistically significant change).

**Table 2:** Top ten downmethylation peaks and their corresponding genes (P7 *vs.* P1).

Chromosome	TxStart	TxEnd	Gene name	Fold change
7	131391143	131391193	**Pstk ↑**	81.6
2	53218067	53218117	**Arl6ip6**	60.7
X	71315056	71315181	**Mtm1 ↓**	58.0
12	69963717	69963767	**Atl1**	27.7
2	122447916	122452011	**Slc28a2**	20.6
4	139962219	139962419	**Klhdc7a ↑**	20.0
2	122452036	122452531	**Slc28a2**	19.70
2	23507969	23508019	**Spopl**	18.1
5	149186468	149186518	**Uspl1 ↓**	17.4
17	84184705	84184805	**Zfp36l2**	15

Top ten downmethylation peaks and their corresponding genes (P7 *vs.* P1). Fold change represents methylation peak's degree. Arrow represents up or down regulation of gene expression, Log_2_FC > 1.5, *P* < 0.05 (no arrow means no statistically significant change).

**Table 3:** Top ten upmethylation peaks and their corresponding gene (P28 *vs.* P1).

Chromosome	TxStart	TxEnd	Gene name	Fold change
2	86042835	86043035	**Olfr1033**	59.9
7	74359749	74359849	**Slco3a1 ↑**	28.3
4	139962244	139962419	**Klhdc7a ↑**	26.1
11	55179387	55180723	**Slc36a2**	24.5
19	10450168	10450218	**Syt7 ↑**	21.8
8	83572497	83572547	**Tecr ↓**	20.2
6	24570892	24571067	**Asb15 ↓**	19.5
14	101442734	101442859	**Tbc1d4 ↑**	19.4
12	91633008	91633133	**Ston2 ↑**	19.3
8	83572397	83572472	**Nr1d2**	16.4

Top ten upmethylation peaks and their corresponding genes (P28 *vs.* P1). Fold change represents methylation peak's degree. Arrow represents up or down regulation of gene expression, Log_2_FC > 1.5, *P* < 0.05 (no arrow means no statistically significant change).

**Table 4:** Top ten downmethylation peaks and their corresponding genes (P28 *vs.* P1).

Chromosome	TxStart	TxEnd	Gene name	Fold change
12	27342363	27342463	**Sox11 ↓**	48.5
3	96240098	96240173	**H2ac19**	17.7
3	96240092	96240192	**Gm20632**	16.7
3	96268599	96268899	**Gm20628**	13.3
11	62648384	62648484	**Lrrc75a**	13.1
7	143460986	143461050	**Cdkn1c ↓**	12.8
4	109666053	109666103	**Cdkn2c**	11.9
3	96268653	96268903	**H3c13 ↓**	11.4
13	23533930	23534005	**H2ac10 ↓**	11.1
13	23574380	23574680	**H2ac7 ↓**	10.8

Top ten downmethylation peaks and their corresponding genes (P28 *vs.* P1). Fold change represents methylation peak's degree. Arrow represents up or down regulation of gene expression, Log_2_FC > 1.5, *P* < 0.05 (no arrow means no statistically significant change).

## Data Availability

The data used to support the findings of this study are available from the corresponding author upon request.

## Abbreviations

BP: Biological process
CC: Cellular component
CDS: Coding sequences
3′UTR: 3′-Untranslated regions
CMs: Cardiac cardiomyocytes
DEGs: Differentially expressed genes
FC: Fold change
FE: Fold enrichment
IRI: Ischemia reperfusion injury
MI: Myocardial infarction
MF: Molecular function
RIN: RNA integrity number.
